# Novel tyrosinase inhibitory peptide with free radical scavenging ability

**DOI:** 10.1080/14756366.2019.1661401

**Published:** 2019-09-08

**Authors:** Zhiwei Shen, Yujiao Wang, Zhen Guo, Tingyuan Tan, Yi Zhang

**Affiliations:** a Key Laboratory of Interfacial Physics and Technology, Shanghai Institute of Applied Physics, Chinese Academy of Sciences, Shanghai, China;; b University of Chinese Academy of Sciences, Beijing, China;; c Zhangjiang Lab, Shanghai Advanced Research Institute, Chinese Academy of Sciences, Shanghai, China

**Keywords:** Melanin, radical scavenging, tyrosinase inhibitory peptide

## Abstract

Tyrosinase is a key enzyme involved in melanin synthesis. Therefore, various tyrosinase inhibitors have been screened by researchers in recent years. In the present study, we discovered a novel tyrosinase inhibitor, a peptide ECGYF (named EF-5), with free radical scavenging ability. The effect of tyrosinase inhibition by EF-5 was stronger than that of arbutin and glutathione, when analyzed both *in vitro* (IC50: 0.46 mM) and *in vivo*. The UV-Vis absorption and circular dichroism spectroscopies indicated that EF-5 interacted with tyrosinase in a different way as that of glutathione. The results of molecular docking showed that the binding between EF-5 and tyrosinase was determined majorly by hydrogen bonds and hydrophobic interactions. EF-5 had also retained its ability to scavenge both hydroxyl and superoxide radicals *in vitro* and was found to be nontoxic to cells, as revealed by the MTT assay. These features suggested that the EF-5 peptide may serve as a safe and effective alternative as a tyrosinase inhibitor.

## 1. Introduction

Tyrosinase is a copper-containing enzyme catalyzing a monophenol (tyrosine) or o-diphenol (L-DOPA) to o-quinone, which later forms true melanin through spontaneous reactions such as cyclization, decarboxylation and oxidative polymerization^1^. Additionally, o-quinone also can form brown melanin when a thiol group participates in the reaction. Both types of melanin are produced by a classical Raper-Mason pathway^1–3^. Thus, tyrosinase plays a critical role in melanin biosynthesis^4–6^.

Melanin is a natural pigment present widely in living organisms. The primary role of melanin is to reduce the skin damage caused by ultraviolet (UV) radiation. On one hand, it serves as a physical barrier and absorbent filter that scatters and limits the penetration of UV radiation^7^. On the other hand, it also can scavenge harmful free radicals induced by the UV radiation^7–9^. However, excessive accumulation and overproduction of melanin can result in the development of physiological abnormalities such as pigment spots, chloasma, freckles, age spots, and even melanoma, as well as neurodegeneration-associated diseases such as the Parkinson’s disease^10–12^. Furthermore, overproduction of melanin in vegetables accelerates corruption, leading to the loss of nutrients and economic wastage^13^^,^^14^.

In order to avoid the overproduction of melanin in living organisms, tyrosinase inhibitors have attracted great interest in the fields of medicine, food, and cosmetics^15^. Many tyrosinase inhibitors such as ascorbic acid, hydroquinone, kojic acid, arbutin, sulfite etc. have been reported previously^5^. However, the adverse side effects associated with their use have led to restrictions in their applications^16^. For instance, arbutin and hydroquinone have been reported to cause contact dermatitis and exogenous ochronosis, respectively^17–19^; benzoquinone, the metabolite of hydroquinone, was found to be cytotoxic to hepatocytes and melanocytes, and may also be carcinogenic^20^^,^^21^; kojic acid, also, has demonstrated carcinogenicity^22^^,^^23^. Currently, several countries have officially banned the use of hydroquinone and kojic acid for skin treatments^24^. Therefore, it is necessary to develop safe tyrosinase inhibitors. Additionally, when tyrosinase inhibitors reduce the production of melanin, the levels of free radicals may increase since melanin is a natural free radical scavenger in living organisms. Ideally, a good tyrosinase inhibitor should also have a free radical scavenging ability.

Tyrosinase inhibitors from natural sources are believed to be safe and are considered to have many potential applications. Some proteins and peptides from milk, silk, sunflower, and honey have shown tyrosinase inhibitory activity, and several peptides inhibiting tyrosinase have been used to treat skin disorders^25^^,^^26^. Practically, short-sequenced peptides with a strong activity for tyrosinase inhibition should be welcome since they are economically viable^27^.

In this paper, we have reported a novel pentapeptide with tyrosinase inhibitory activity and free radical scavenging ability. The peptide, ECGYF (named EF-5), consists of a short sequence of the protein midasin found in *Vigna*. The activity of EF-5 to inhibit tyrosinase was greater than that of glutathione and arbutin, but was nontoxic to the melanocytes. Particularly, its ability to scavenge free radicals was as strong as that of glutathione. These results have indicated that the EF-5 peptide is an ideal tyrosinase inhibitor that can be used in cosmetics as a safe and efficient melanin scavenger.

## 2. Materials and methods

### 2.1. Materials

The peptide EF-5 was synthesized using the standard Fmoc solid phase synthesis method, as per the procedure described previously^28^. The HPLC and mass spectra of EF-5 are shown Supplemental Figure S1 and Supplemental Figure S2, respectively. Tyrosinase extracted from mushrooms was purchased from Sigma-Aldrich. Arbutin was purchased from the Tokyo Chemical Industry. All other chemicals used were of the reagent grade and purchased from Sigma-Aldrich (USA), unless specified otherwise.

**Figure 1.**
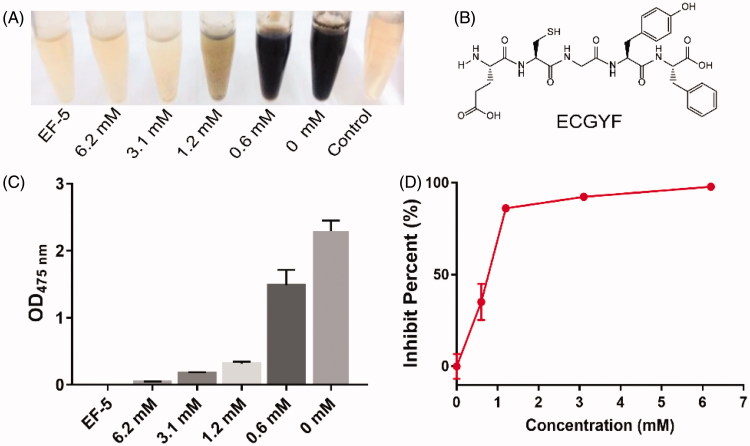
Inhibition ability of the peptide EF-5 on tyrosinase. (A) A picture of the tyrosine-saturated PBS solutions after mixing with tyrosinase (1 KU/ml) and EF-5 (concentrations varied in 0–6.2 mM). Control group contains only tyrosinase. EF-5 group contains only EF-5 (6.2 mM) and tyrosinase (1 KU/ml) for 24 h. (B) Molecular structure of peptide EF-5. (C) The absorbance at 475 nm of the solutions shown in (A). The absorbance of tyrosinase was subtracted from each group. *N* = 5. (D) The ratio of tyrosinase activity in the melanin production inhibited by EF-5 plotted vs EF-5 concentration. *N* = 3.

**Figure 2.**
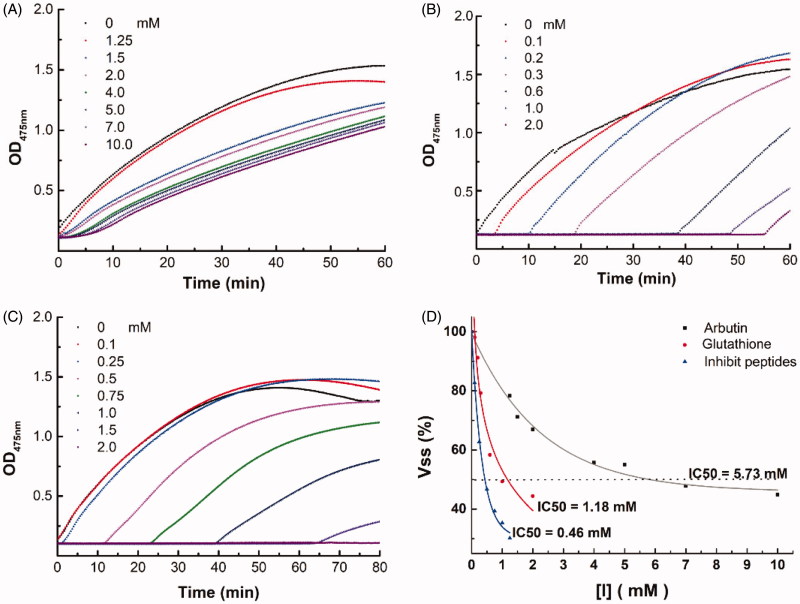
(A–C) Evolution of OD value at 475 nm in the tyrosine solutions mixed with tyrosinase and its inhibitors, Arbutin (A), Glutathione (B), or EF-5 (C). (D) The plot of the percentage of enzyme activity (Vss) vs the tyrosinase concentration [I]. IC50 of the inhibitors were directly obtained from the plots.

### 2.2. Tyrosinase activity inhibition assay

The inhibition assays performed to determine the activities of mushroom tyrosinase were carried out by spectrophotometrically measuring the rate of melanin formation. Equal amount of tyrosinase was added to a phosphate buffer solution (PBS) saturated with tyrosine (2.5 mM), to which, solutions of EF-5 at different concentrations were sequentially added. After incubation for 24 h at 37 °C, the visible light absorbance was measured at 475 nm using a spectrophotometer (U-3010, HITACHI, Japan). All measurements were repeated thrice. The value of light absorbance of the corresponding group in the absence of tyrosinase was subtracted. The rate of enzyme inhibition was calculated using following formula:

(1) $$\text{Inhibition percentage \%}=\left[1-\frac{A-B}{A-C}\right]\times 100$$

where *A*, *B* and *C* are the values of light absorbance in the absence and presence of the peptide inhibitor, and tyrosinase dissolved in PBS, respectively.

### 2.3. Comparison with the tyrosinase inhibition rate of glutathione and arbutin

The maximum reaction rates of tyrosinase at different concentrations of EF-5, glutathione, and arbutin, were measured, and the half maximal inhibitory concentrations (IC50) of the inhibitors were compared. Before conducting the experiment, all the solutions, 96-well plates, and tips were heated at 37 °C. Equal volumes of tyrosine solutions (final concentration of 2.5 mM) were added to the 96-well plates, mixed with different concentrations of the inhibitors mentioned above and incubated for 10 min at 37 °C. Then, equal volume of the tyrosinase solution (100 U per well) was added to the 96-well plate using a multichannel pipette, which was placed immediately in a microplate reader under shaking conditions for 30 s. Finally, the light absorbance value at 475 nm was recorded at 37 °C continuously for 2 h with intervals of 20 s. The maximum reaction rate of tyrosinase at each concentration of the inhibitor was calculated.

### 2.4. UV-Vis absorption spectra of tyrosinase

The UV absorption of tyrosinase, EF-5 (1.5 mM) and glutathione (3 mM) in PBS was measured by recording the absorption spectra at 250–400 nm using a U-3010 spectrophotometer (HITACHI, Japan).

### 2.5. CD Spectroscopy

The secondary structures of EF-5, glutathione, tyrosinase, and their mixtures were recorded over the wavelength range of 200–260 nm using far-UV CD spectroscopy (Applied Photophysics, UK). The CD measurements were performed at 293.15 K with a bandwidth, step interval and scanning speed of 1.0 nm, 1 nm, and 50 nm min^−1^, respectively.

### 2.6. *In vitro* free radical scavenging effect

#### 2.6.1. Superoxide anion radical scavenging

Auto-oxidation of pyrogallol was employed to determine the free radical scavenging ability of EF-5^29^^,^^30^. Tris-HCl (0.1 M, pH 8.2) at a volume of 750 µl was mixed with 200 µl PBS solutions of EF-5 or glutathione at different concentrations at room temperature, and then 70 µl pyrogallol (50 mM dissolved in deionized water) or deionized water (as sample blank) was added to the mixture. After incubation for 4 min in the dark, the reaction was stopped by adding 20 µl HCl (8 M) to the reaction system. Measurement of absorbance at 320 nm was carried out using a spectrophotometer (U-3010, HITACHI, Japan). The superoxide anion radical scavenging effect was calculated using the following equation:

(2) $$\text{Scavenging effect }\left(\%\right)=[1-\frac{{(A}_{\mathrm{sample}}-A_{\mathrm{sample}\;\mathrm{blank}})}{\left(A_{\mathrm{control}}-A_{\mathrm{blank}}\right)}]\times 100$$

#### 2.6.2 Hydroxyl radical scavenging

The hydroxyl radicals generated by the Fenton reaction^31^ were employed to determine the scavenging ability of EF-5 or glutathione^29^. Firstly, 250 µl FeSO_4_ (10 mmol/L) was mixed with 250 µl 1, 10-phenanthroline (10 mmol/L) and 200 µl Tris-HCl (50 Mm, pH 7.4). Then, 100 µl H_2_O_2_ (1.5 mmol/L) or deionized water (blank group), and 300 µl EF-5 in PBS with different concentrations or deionized water (control group) was added, respectively. The mixtures were incubated for 30 min in a water bath at 37 °C. The absorbance at 510 nm was recorded by a U-3010 spectrophotometer (HITACHI, Japan). The scavenging ability on hydroxyl radicals was calculated using the Equation (3), which is as follows:

(3) $$\text{Scavenging effect }\left(\%\right)=[1-\frac{{(A}_{\mathrm{sample}}-A_{\mathrm{blank}})}{{(A}_{\mathrm{control}}-A_{\mathrm{blank}})}]\times 100$$

### 2.7. Cell culture

A human melanoma cell line (A375) was provided by the Stem Cell Bank, Chinese Academy of Sciences. The A375 cell line was cultured in DMEM media containing 10% fetal bovine serum and 1% penicillin/streptomycin in an incubator at 37 °C and an atmosphere containing 5% CO_2_^32^.

### 2.8. Intracellular tyrosinase activity inhibition assay

The A375 cells were seeded at a density of 1 × 10^5^ cells/well in a 6-well plate and cultured for 12 h. Then, they were treated with different tyrosinase inhibitors (final concentration 2.5 mM) and further cultured for 60 h. The cells were then separated using a 0.25% trypsin-EDTA solution, centrifuged (1000 rpm, 3 min) and washed twice with PBS. After adding 0.5 ml NaOH (1 M), the cells were disrupted by sonication, and incubated at 50 °C for 10 min. Finally, the absorbance at 475 nm was detected using a microplate reader (VersaMax Microplate Reader)^33^.

### 2.9. MTT assay

The A375 cells were seeded at a density of 1 × 10^4^ cells per well in a 96-well plate. They were incubated for 24 h at 37 °C under an atmosphere containing 5% CO_2_. Then, different tyrosinase inhibitors were added into the culture media. After incubation for 24 h, the cell viability was measured using the MTT assay^34^, which was based on the conversion of MTT to formazan crystals by mitochondrial dehydrogenases. Briefly, the cell cultures were incubated with a 20 µl MTT solution (5 mg/mL) for 4 h at 37 °C. The culture supernatant was carefully removed from the well, and 150 µl DMSO was added followed by shaking for 10 min to dissolve the formazan crystals. Absorbance at 492 nm was measured using a microplate reader (VersaMax Microplate Reader)^35^. Cell viability of the control group in which tyrosinase was replaced by PBS was defined as 100%^36^.

### 2.10. Molecular docking investigation

Molecular docking of tyrosinase and EF-5 was investigated using the program AutoDock 4.0. The 3 D map of EF-5 peptide docking with the tyrosinase activity center was illustrated using PyMOL, and the 2 D map of the docking model was illustrated using the software LigPlot 2.0. The crystal structure of tyrosinase (PDB ID: 2Y9X) was obtained from the RCSB Protein Data Bank (https://www.rcsb.org/structure/2Y9X).

## 3. Results and discussion

### 3.1. Inhibition of the tyrosinase activity by EF-5 *in vitro*

Since tyrosinase is a key enzyme in the synthesis of melanin, we tested the ability of EF-5 to inhibit tyrosinase-catalyzed melanin production. As shown in Figure 1, when PBS solutions saturated with tyrosine were mixed with different concentrations of tyrosinase (1 KU) and EF-5, a negative correlation was observed between the production of melanin and the concentration of EF-5, indicating that EF-5 is able to inhibit tyrosinase significantly. In an extreme case, when the concentration of EF-5 had reached 6.2 mM, the activity of tyrosinase was inhibited almost completely (Figure 1(D)).

Previously, it has been reported that combination of the sulfhydryl group of glutathione with o-quinones would form phaeomelanins and trichochromes in a low speed reaction due to the catalytic activity of tyrosinase^2^ and melanin would be produced continuously as the thiol compound is consumed. However, although the EF-5 contains a sequence of glutathione (Figure 1(B)), in our experiments, no change in the absorbance values of the solutions in the presence of EF-5 was observed for long period, e.g., 12 m (compare Figure 1(C) with Supplemental Figure S3), which indicates that the EF-5 peptide is effective and can retain its ability to inhibit the tyrosinase activity for a long time.

**Figure 3.**
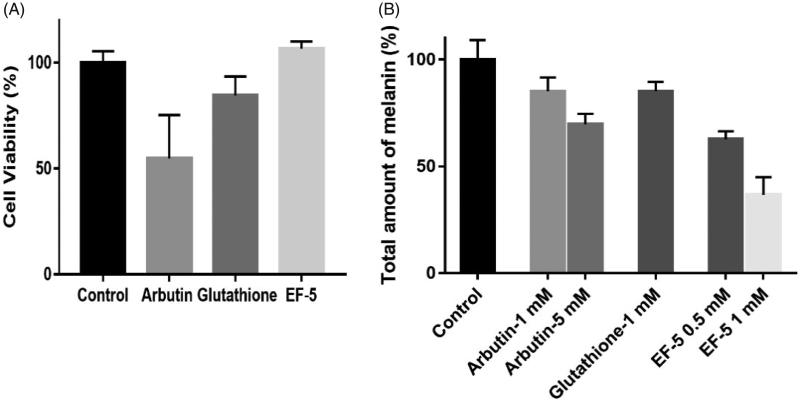
(A) Effect of inhibitors on cell viability. The concentrations of all inhibitors were 2.5 mM in PBS. (B) Comparison on melanin production in melanocyte with the treatment of inhibitors. *N* = 3. The error bars represent means ± SD

Since the IC50 values of the tyrosinase inhibitors change with the purity of the mushroom tyrosinase^37^, it is difficult to directly evaluate the absolute IC50 value of EF-5. In this study, we tested and compared the IC50 of EF-5 with known tyrosinase inhibitors such as arbutin and glutathione. As shown in Figure 2, the IC50 of arbutin, glutathione, and EF-5 were 5.73 mM, 1.18 mM, and 0.46 mM, respectively, indicating that the inhibition ability of EF-5 was much stronger than that of the others. Using the IC50 of arbutin as a reference, it was determined that the ability of EF-5 to inhibit tyrosinase was also stronger than some antimelanogenic peptides reported previously, which were obtained from the enzymatic hydrolysis of rice brain proteins^38–39^. EF-5 and glutathione contain the sulfhydryl group, which may significantly increase the lag phase of the catalytic reaction of tyrosinase (comparing Figure 2(B) and Figure 2(C) with Figure 2(A)).

### 3.^2^. Inhibition of the tyrosinase activity by EF-5 on melanocytes

Tyrosinase derived from mushrooms is present in the cytoplasm, which is different from the mammalian tyrosinase present on the membrane of melanin bodies^40^^,^^41^. Therefore, the effects of tyrosinase inhibitors within and outside melanocytes may be different. We compared the effects of EF-5, glutathione and arbutin on cell viability and melanin synthesis in melanoma cells (A375). The results indicated that the inhibitory effect of arbutin on the viability of melanoma cells is significant, while that of glutathione and EF-5 is not (Figure 3(A)). Arbutin, at a concentration of 2.5 mM, was also found to inhibit proliferation of the cells while the others did not (Supplemental Figure S4).

**Figure 4.**
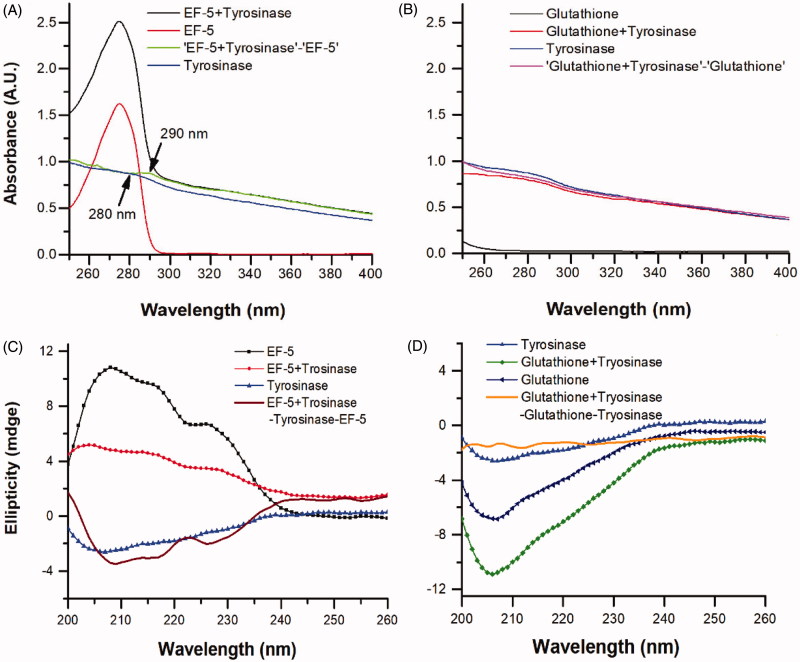
The UV-Vis absorption spectra of tyrosinase in the presence of (A) EF-5 and (B) glutathione, and the CD spectra of tyrosinase in the presence of (C) EF-5 and (D) Glutathione. The concentrations of EF-5, glutathione and tyrosinase were 0.2 mg/ml.

Our results indicated that, when at concentrations of 1 mM, the melanin inhibitory effects of arbutin and glutathione were similar, while that of EF-5 was twice compared to the other two. The melanin inhibitory effects of arbutin and EF-5 at concentrations of 5 mM and 0.5 mM, respectively are similar (Figure 3(B)). It should be noted that, in our experiments, the total concentration of cells in the arbutin (5 mM) group was much lesser than that in the EF-5 (0.5 mM) group (Supplemental Figure S4); therefore, the ability of EF-5 to inhibit tyrosine is likely to be better in the melanoma cells.

### 3.3. Conformational change of tyrosinase in the presence of EF-5

We investigated the effects of EF-5 and glutathione on the structure of tyrosinase. The absorption spectra of tyrosinase in the presence of EF-5 (or glutathione) were obtained by subtracting the corresponding spectra of EF-5 (or glutathione) from that of the mixture. It is expected that the spectra of tyrosinase in the presence of EF-5 (or glutathione) should be consistent with the spectra of tyrosinase alone if no interaction is taking place between tyrosinase and EF-5 (or glutathione)^42^. When EF-5 was added to a tyrosinase solution, the UV absorption peak was found to be red-shifted from 280 nm to 290 nm (see blue and navy lines in Figure 4(A)), suggesting a decrease in the surface hydrophobicity of tyrosinase^42^. However, such an effect on the absorption peak of tyrosinase (see blue and green lines in Figure 4(B)) was not observed with glutathione.

CD spectroscopy is a useful technique for studying protein–protein interactions in a solution^43^. The CD spectra were recorded in order to further study a change in the structure of tyrosinase in the presence of EF-5 or glutathione, (Figure 4(C,D)). The spectrum of the mixture of EF-5 and tyrosinase increases at 208, 218 and 226 nm (Figure 4(C)), indicating an increase in the amount of α-helix and β-turns^44^. Contrarily, the spectrum of the mixture of tyrosinase and glutathione does not show such changes (Figure 4(D))^44^. These results suggest that EF-5 indeed binds to and interacts with tyrosinase and changes its conformation. Previous researches have reported that the thiol group of glutathione chelates the copper ions in the active site of the tyrosinase and interacts with it^2^^,^^27^. Therefore, our results have indicated that EF-5 did not interact with tyrosinase simply via the sulfhydryl of the cysteine residue as in glutathione.

### 3.4. Molecular docking

**Figure 5.**
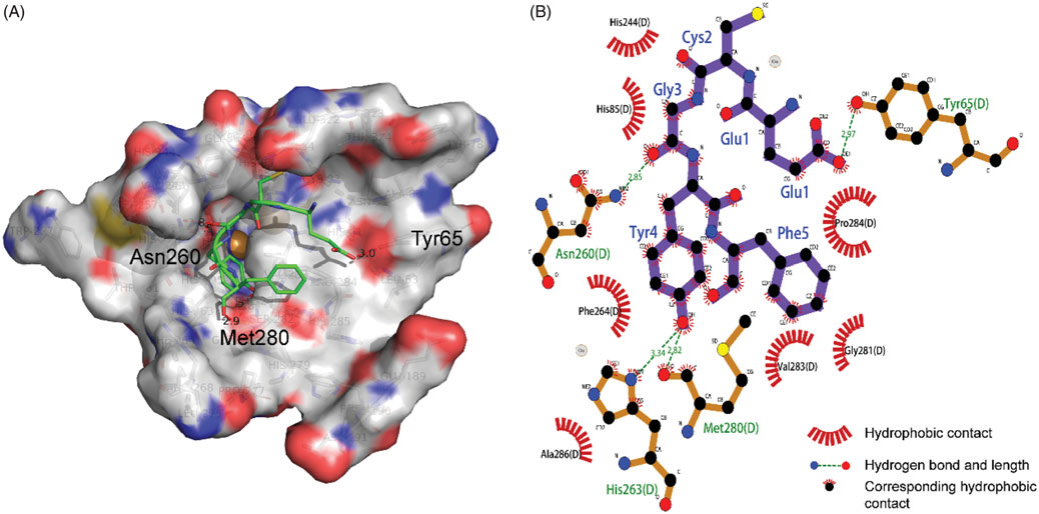
Molecular docking model of tyrosinase and EF-5. (A) The 3 D map of EF-5 docking in the tyrosinase activity center. (B) The 2 D projection of EF-5 and tyrosinase docking model. The image was generated in LigPlot.

Tyrosinase is a copper-containing enzyme, with two copper ions in its active center chelated by six histidine residues (His61, His85, His94, His259, His263, and His296), which are directly involved in different catalytic activities^45–46^. A molecular docking study was carried out to reveal the interaction between the tyrosinase activity pocket and EF-5 peptide. The most probable interaction model of tyrosinase binding EF-5, with a binding energy of −2.15 kcal mol^−1^, is shown in Figure 5. In the central region of its activity, EF-5 may interact with tyrosinase through hydrogen bonds at the residuals Tyr65, Asp260, His263, and Met280. A hydrogen bond with a bond length of 2.82 Å forms between the oxygen atom of the carbonyl group in residue Met280 and the hydrogen atom of the phenolic hydroxyl group in the tyrosine residue. The Met280 residue plays an important role in the tyrosinase activity, and many of the reported tyrosinase inhibitors inhibit its activity by forming a hydrogen bond with this residue^27^^,^^42^^,^^47^. The residue His263 chelates directly with the copper ions. When the oxygen atom of the tyrosine residue on EF-5 bonds with the hydrogen atom of His263, it will affect the activity of the enzyme, thereby inhibiting the production of melanin. The benzene ring of the tyrosine of the EF-5 peptide undergoes strong hydrophobic interactions with Phe264 and Pro284, which may block the hydrophobic pocket of tyrosinase, thereby reducing its hydrophobicity^48^.

### 3.5. Reactive oxygen species (ROS) scavenging effect of EF-5 *in vitro*

**Figure 6.**
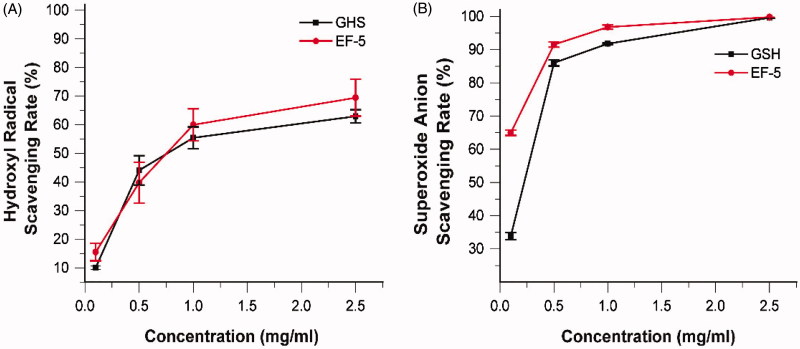
Scavenging effect (%) of EF-5 and glutathione on hydroxyl radical (A) and superoxide radical (B). *N* = 3.

Melanin scavenges free radicals induced by the UV radiation, and functions as an antioxidant in nature^8^^,^^49–51^. Therefore, when a tyrosinase inhibitor reduces the production of melanin, its ROS scavenging ability should be compensated. Antioxidants play a synergistic inhibitory role in tyrosinase inhibition^52^. Glutathione is involved in defense against ROS, and the reducing thiol group provides the electrons required for free radical pairing. Since EF-5 contains the same sequence as glutathione, the free radical scavenging ability of EF-5 was tested and compared with that of glutathione. The results indicated that, taken together, they demonstrated similar ROS scavenging ability (Figure 6). However, the ability of EF-5 to scavenge the hydroxyl radical is greater than that of glutathione at low concentrations (Figure 6(A)). At high concentrations, the rate of superoxide anion radical scavenging of EF-5 is greater than that of glutathione (Figure 6(B)).

## 4. Conclusions

In summary, we have reported a pentapeptide, EF-5, which is not only effective in inhibiting the tyrosinase activity but also possesses free radical scavenging ability. The IC50 of EF-5 was found to be about 1/12 and 1/4 that of arbutin and glutathione, respectively. *In vivo* studies have also indicated that the ability of EF-5 to inhibit tyrosinase is very remarkable tyrosinase inhibition ability with the highest cell viability. The UV-Vis absorption spectra, CD spectra and molecular docking have suggested that the EF-5 peptides might induce conformational changes in tyrosinase and interact with it differently than with glutathione. Additionally, EF-5 showed strong reactive free radical scavenging properties due to its glutathione-like sequence, which compensates at least in part to the free radical scavenging ability of melanin. These results have indicated that EF-5 may be used as a potential tyrosinase inhibitor in cosmetics or to treat skin disorders.

## Supplementary Material

Supplemental Material
