# Supplementary material for: Novel tyrosinase inhibitory peptide with free radical scavenging ability
Source: J Enzyme Inhib Med Chem. 2019 Sep 8;34(1):1633–40. doi: 10.1080/14756366.2019.1661401 (PMC6746264; doi:10.1080/14756366.2019.1661401)
Supplement: Supplemental Material [file IENZ_A_1661401_SM1720.pdf]

Supplementary Material

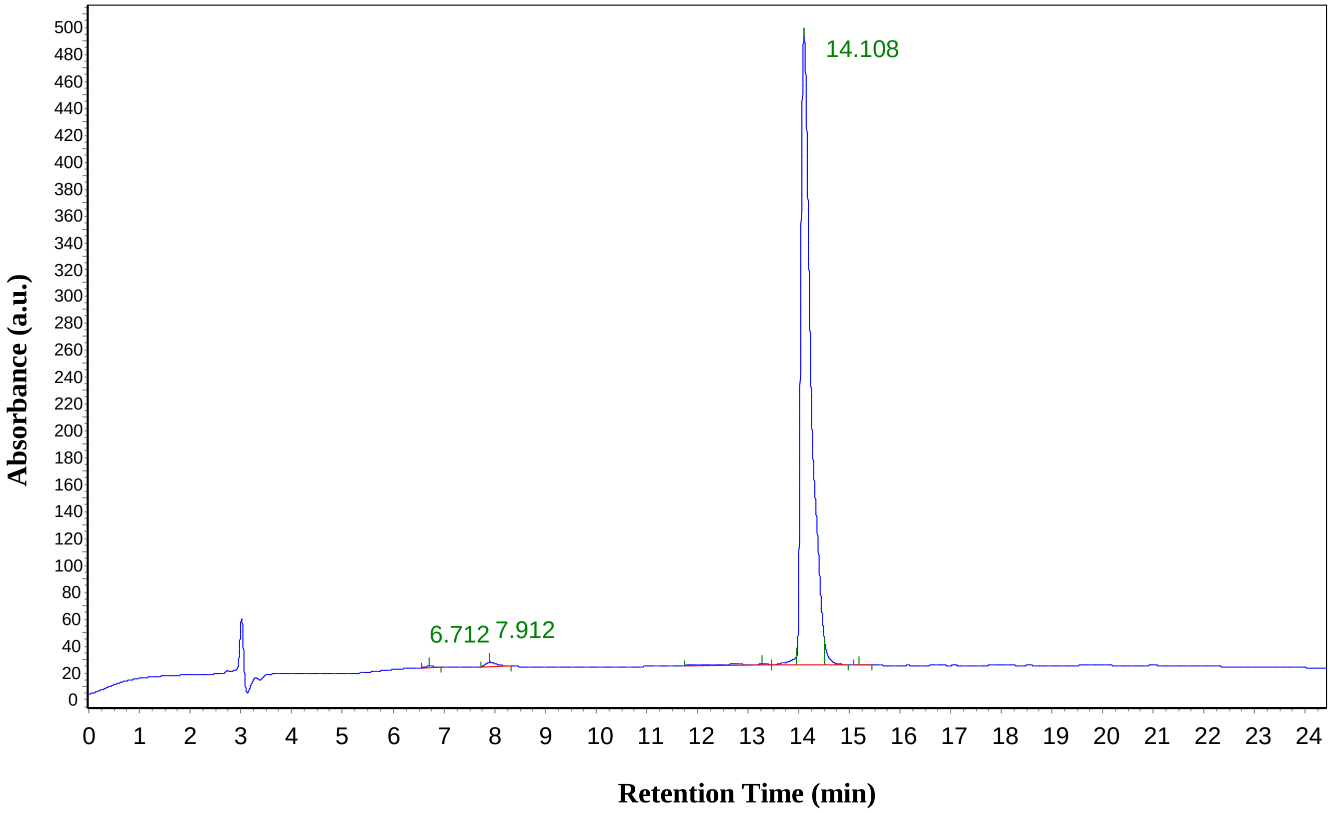

Fig. 1S. A Chromatogram of the transitions of the peptide EF-5. The peak at retention time of 14.108 min is the product peak. Column eluate was monitored at 220 nm and a flow rate of 1 ml/min with 0.1% Trifluoroacetic Acid in 100% Acetonitrile. EF-5 has a purity of 96.25%.

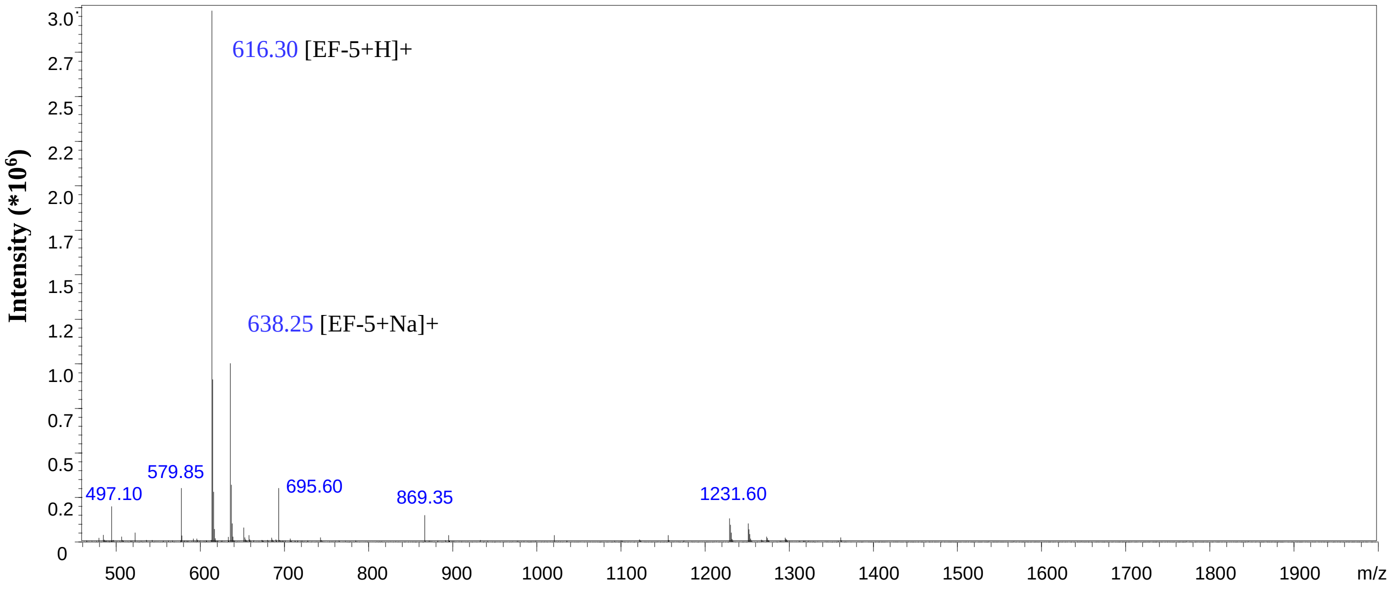

Fig. S2. ESI/Mass spectrometry indicating that the peptide EF-5 has a molecular weight of 615.3.

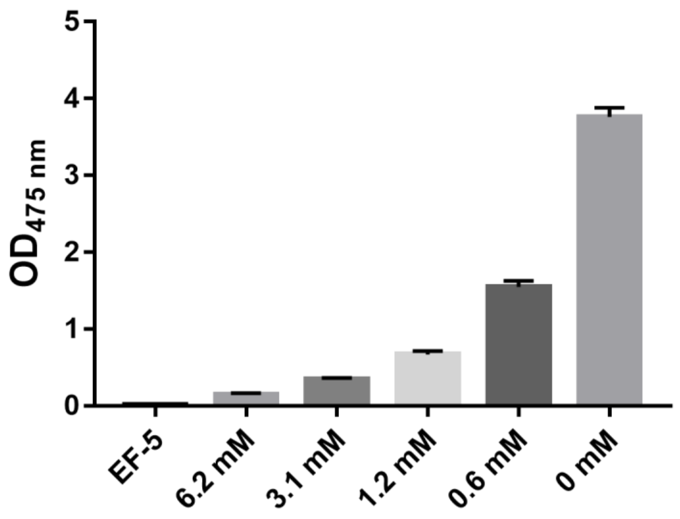

Fig. S3. The OD values at 475 nm of the solutions shown in Fig. 1A after storage for 12 months. Error bars represent S.D. N=3.

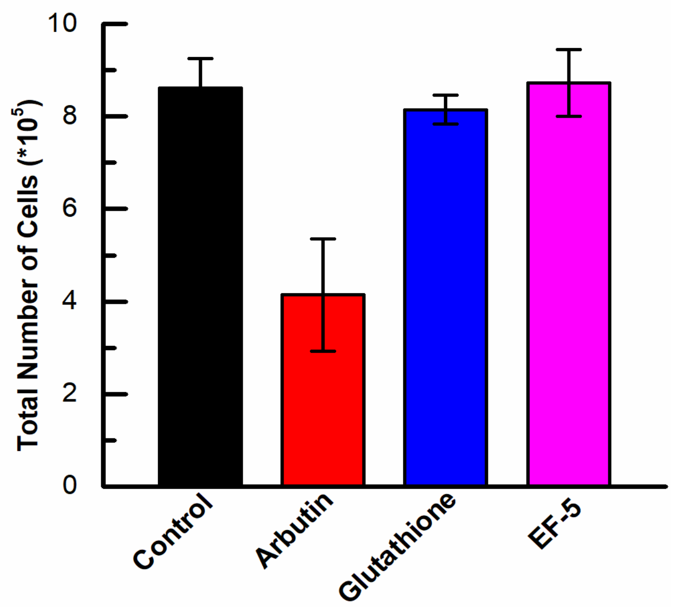

Fig. S4. Total number of proliferated melanoma cells in the presence of different tyrosinase inhibitors. The concentration of each inhibitor is 2.5 mM. Error bars represent S.D. N=3.
